# “…*We never considered it important…”*: a qualitative study on perceived barriers on use of non-pharmacological methods in management of labour pain by nurse-midwives in eastern Tanzania

**DOI:** 10.1186/s12912-024-02187-2

**Published:** 2024-07-29

**Authors:** Dorkasi L. Mwakawanga, Nathanael Sirili, Victor Z. Chikwala, Lilian T. Mselle

**Affiliations:** 1 Department of Community Health Nursing, School of Nursing, Muhimbili University of Health and Allied Sciences, Dar es Salaam, Tanzania; 2 Department of Development Studies, School of Public Health and Social Sciences, Muhimbili University of Health and Allied Sciences, Dar es Salaam, Tanzania; 3 Department of Clinical Nursing, School of Nursing, Muhimbili University of Health and Allied Sciences, Dar es Salaam, Tanzania

**Keywords:** Labour pain management, Pharmacological methods, Non-pharmacological methods, Nurse-midwives, Barriers, Tanzania

## Abstract

**Background:**

A significant number of women experience labour without effective pain management and thus suffer from unbearable labour pain to the extent they term labour as the most agonizing event in their lives. Unresolved labour pain can lead to stress, fear, and confusion, which may compromise placental perfusion and lead to birth asphyxia. Although various pharmacological and non-pharmacological labour pain management methods exist, the use of non-pharmacological methods (NPMs) to manage labour pain has remained low in low-resource settings. This paper explored the barriers for using NPMs to manage labour pain by nurse-midwives in eastern Tanzania.

**Methods:**

We conducted an exploratory qualitative study with 18 nurse-midwives purposefully recruited from the labour wards of two selected district hospitals in eastern Tanzania. Qualitative content analysis guided the data analysis.

**Results:**

Two categories illustrating barriers to using NPMs were generated: individual-level and institutional-level barriers. Individual-level barriers include (i) limited competencies of nurse-midwives on the use of NPMs for managing labour pain, (ii) inadequate exposure to labour pain management practices, (iii) misconceptions about labour pain relief, and (iv) a lack of opportunities for knowledge acquisition. The institutional barriers include (i) a critical staff shortage amidst many clients and (ii) an unfavourable healthcare facility environment.

**Conclusion:**

The implementation of NPMs for labour pain management by nurse-midwives in eastern Tanzania faces several institutional and individual barriers. We recommend addressing both supply- and demand-side barriers. Strengthening nurse midwives’ competencies in NPMs adoption and use and improving the facility environment to ensure privacy during labour can be a starting point for addressing supply-side issues. We recommend dispelling myths and misconceptions through health promotion education to address demand-side barriers.

## Introduction

Labour pain management is an essential component in providing respectful maternity care and satisfying the needs of women who access childbirth services in healthcare facilities [1, 2]. A significant number of women experience labour without proper pain management, resulting in unbearable pain that is described as the most agonizing experience of their lives [3]. Prompt and effective labour pain relief is recommended to be offered based on the woman’s preferences, culture, and needs [4]. Providing proper pain relief not only contributes to humanizing childbirth but also yields significant physiological and psychological benefits to both the mother and the baby [5]. Unresolved labour pain can impair placental perfusion, resulting in late decelerations, foetal hypoxia and distress [6]. It can also induce fear, stress, and confusion to the mother [7].

Pharmacological and non-pharmacological methods (NPMs) are the main available options for labour pain management [8]. The pharmacological methods include oral tablets, inhalation analgesia, intravenous and intramuscular opioids (pethidine or diamorphine), and epidural or spinal anaesthetic analgesia [9]. Pharmacological methods for labour pain management are costly and are associated with side effects such as maternal nausea, vomiting, drowsiness, hypotension, urine retention, nerve damage, and prolonged labour [1, 10–12]. Prolonged labour may predispose a woman to labour augmentation, instrumental delivery, and caesarean section delivery [13]. These delivery methods may result in maternal complications, including rupture of the uterus, postpartum haemorrhage, sepsis, and maternal and perinatal deaths [14].

The NPMs include the use of birth preparation classes, breathing exercises, massages, music, warm showers, birth balls, and transcutaneous electrical nerve stimulation (TENS) during the early stages of labour [4, 15]. Despite the wide variation in NPMs [16], it is encouraged to use any of the NPM approaches to relieve labour pain. NPMs are considered simple, cost-effective, manageable, induce calm, and help women to cope with labour pain [1]. Moreover, using NPMs for labour pain relief alleviates stress, tends to lessen urgent and operative interventions, and makes women favour a natural birth experience. [17].

The provider’s willingness, knowledge, perceptions, attitude, practice, and beliefs influence the use of NPMs in managing labour pain [18, 19]. The familiarity with NPMs and professional experiences with deliveries conducted may also impact the use of NPMs for managing labour pain [20]. Furthermore, the literature indicates that the availability of policy, supportive infrastructure, and resources influences the utilization of NPMs for labour pain management [20, 21]. In many low- and low-middle-income countries, including Tanzania, women in labour are mainly managed by nurse-midwives; thus, they are a good target for implementing NPMs for labour pain management [10, 22–24]. A nurse-midwife in Tanzania is a professional who has completed a 3- or 4-year training program in midwifery and nursing at an accredited college or university and holds a license to practice [25].

In high-income countries, pharmacological labour pain management is a fundamental part of the labour process, and women have access to a range of pain relief options for labour and birth [18, 26, 27]. Conversely, in low- and low-middle-income countries, people often disregard labour pain management, viewing labour pain as a natural process that women should be able to cope with [18]. There are no clear labour pain management guidelines or policies [28, 29], but the existing intrapartum and respectful maternity care guidelines promote using labour pain relief approaches such as psychological support [30]. Several factors, including limited awareness, misunderstandings regarding safety and acceptability, and the availability of pain relief options, contribute to the limited use of pharmacological methods for labour pain relief in these countries [31, 32]. Despite the limited use of pharmacological labour pain management methods, there is paucity of information regarding the use of NPMs for labour pain management. This study explored the barriers for using NPMs to manage labour pain as reported by nurse-midwives from two selected district hospitals in eastern Tanzania.

## Materials and methods

### Study design

We conducted an exploratory qualitative study using in-depth interviews to explore the barriers to using NPMs to manage labour pain at two district hospitals in eastern Tanzania. Exploratory qualitative research is designed to shed light on the diverse manifestations of a phenomenon and its underlying processes [33]. Therefore, we deemed this design appropriate for in-depth exploration to gain a comprehensive understanding of the barriers to using NPMs in managing labour pain.

### Study area

We conducted this study in two government-owned district hospitals in Dar es Salaam City and the Pwani region. Dar es Salaam city and the Pwani region are located in the eastern part of the Tanzanian mainland, along the Indian Ocean coastal belt. Dar es Salaam is the biggest commercial city, with the largest port serving as the country’s economic hub; it denotes population size and economic activities. The Pwani region has a population of 2,024,947, of which 80 per cent depend on agriculture as a means of livelihood [34]. Other sources of economic activity in the Pwani Region include fisheries, industry, mining, and tourism. A network of roads serves the region well, linking the rural population clusters and facilitating easy communication with the surrounding areas. We purposely chose one district hospital in Dar es Salaam to represent the urban area and one from the Pwani region to represent the rural area.

The pyramidal structure of Tanzania’s healthcare system includes three levels: the primary level, comprising district hospitals, health centres, and dispensaries; the secondary level, encompassing regional referral hospitals; and the tertiary level, encompassing zonal, specialized, consultant, and national hospitals [35]. District hospitals function as the primary referral level, providing comprehensive emergency obstetric and newborn care (CEmONC) services [36], with nurse-midwives responsible for the majority of women’s care during labour, delivery and postpartum period [37]. According to the 2022–2023 Demographic Health Survey, 91% of pregnant women from the eastern zone of Tanzania delivered in health facilities [38]. The district hospital`s labour wards can accommodate ten to fifteen clients, although there may only be two to three skilled birth attendants available during each 8-hour shift. In these hospitals, women receive non-pharmacological approaches for labour pain relief, which mainly involve psychological support and exercises such as walking. However, instead of serving as a non-pharmacological method for labour pain relief, these hospitals primarily use exercises to stimulate labour progress through descent. It was believed that the two facilities shared comparable qualities, potentially providing a clear picture of the barriers to using NPMs in urban and rural areas.

### Recruitment of participants

We conducted this study as part of the broader study that aimed to explore the factors influencing the use of NPMs for managing labour pain [29]. Using purposive sampling, we recruited nurse-midwives from two district hospitals in eastern Tanzania. At both facilities, we first approached a nurse-midwife in charge of the labour ward and explained the purpose of our visit. At our request, the nurse-midwife in charge provided a list of nurse-midwives working in the labour ward. The researchers approached each identified participant face-to-face as they were introduced by the in charge, explained the purpose of the contact, and set up an appointment for interview. We contacted a total of 18 midwives, and all agreed to participate in this study. We adopted both intra and inter-saturation of information (i.e., we stopped data collection when no new information was obtained from participants and redundancy was achieved) [39]. For intra saturation, we conducted interviews at each district hospital until no new information obtained. For the inter-saturation, we ensured that across the two district hospitals, there was no new information regarding the barriers to the use of NPMs in managing labour pain that came up with further interviews. As a result, at the first district hospital we achieved saturation with the 8th participant, while at the second hospital we achieved saturation with the 10th participant. We therefore stopped data collection in both district hospitals with a total of 18 participants.

### Data collection process

We developed and used a semi-structured interview guide to conduct in-depth interviews with nurse-midwives from the two hospitals. The guide was developed in English and then translated into Kiswahili; the language spoken by majority in Tanzania. We developed the questions in the guide based on the objectives, researchers’ experience, and literature on using NPMs [26, 31, 40]. The questions and probes focused on the barriers that nurse-midwives face to use NPMs. We conducted the interviews between July and November 2020. To ensure the capture of participants’ accounts [41], we conducted all interviews in Kiswahili. The data collection team included all four authors, of whom the 2nd and 4th are experienced qualitative researchers and mentored the 1st and 3rd authors, who are junior researchers. We interviewed the nurse-midwives at their convenience, using a designated room in the healthcare facility. Interviews lasted between 30 and 60 min. All interviews were audio-recorded, and field notes were taken. The Muhimbili University of Health and Allied Sciences (MUHAS), Research and Ethics Committee (Ref. No. MUHAS-REC-2–2020 − 103) provided the ethical approval to conduct this study. Before each interview, participants provided written informed consent after receiving a briefing about the study`s objectives and the audio recording of the session.

### Data management and analysis

Qualitative content analysis following Graneheim and Lundman [42] guided the analysis of data. Audio-recorded interviews were first transcribed verbatim. All authors repeatedly read the transcribed information and field notes to familiarize themselves with the data and context before coding. The first author developed an initial codebook for data analysis based on our study objectives, interview guide, and the conceptual understanding of barriers to using NPMs to manage labour pain and childbirth care in Tanzania. All authors tested and discussed the codebook to reach consensus before commencing the coding process. [43].

We treated all transcripts as meaningful units. Through data reduction, we created condensed meaning units related to barriers for using NPMs to manage labour pain. We assigned inductive coding to text segments that represented new, non-pre-determined codes. We assigned the new codes as separate codes or as an expansion of the codes found in the initial codebook. All the coders were fluent and native Kiswahili speakers and conducted the coding in Kiswahili. The latter aimed to maintain the originality of the data collected. We then translated the codes into English to illustrate the statements from the participants` accounts. All authors then discussed the initial list of codes before agreeing on the final codes. The management and organization of data were done using the qualitative data analysis NVivo software program. We exported the codes to Microsoft Word for abstraction to subcategories and categories, comparing similarities and differences. We then used succinct quotes translated into English to support the description of categories and subcategories.

## Results

We interviewed 18 nurse-midwives: 10 from Dar es Salaam and 8 from the Pwani region. Table 1 shows the demographic characteristics of the study participants. The median age of nurse-midwives was 37 years (range between 25 and 53). All participants were female. Four had worked in the labour ward for one year, six had worked between 2 and 5 years, and eight had more than 5 years of experience. Most midwives had completed a certificate in nursing (6); 10 had a diploma in nursing and midwifery; and 2 had a bachelor’s degree in nursing or midwifery.

**Table 1:** Demographic characteristics of study participants

Participant’s No.	Age in years	Profession education level	Work experience in years
1	36	Diploma	3
2	42	Certificate	6
3	29	Diploma	1
4	53	Diploma	15
5	37	Certificate	8
6	39	BSc. in Nursing	6
7	36	Diploma	5
8	27	Diploma	1
9	40	BSc. in Midwifery	13
10	25	Certificate	1
11	49	Diploma	10
12	45	Diploma	9
13	30	Certificate	4
14	30	Diploma	1
15	30	Certificate	2
16	48	Diploma	12
17	34	Diploma	3
18	37	Certificate	4

Source: Study participants

Two categories illustrating barriers to using NPMs were generated: individual-level and institutional-level barriers. Individual-level barriers include (i) limited competencies of nurse-midwives on the use of NPMs for managing labour pain, (ii) inadequate exposure to labour pain management practices, (iii) misconceptions about labour pain relief, and (iv) a lack of opportunities for knowledge acquisition. The institutional barriers include (i) a critical staff shortage amidst many clients and (ii) an unfavourable healthcare facility environment attributed to limited privacy and physical space, lack of utilities, pain management guidelines and limited incentives among nurse-midwives (see Table 2**)**

**Table 2:** Summary of findings

Selected codes	Sub-categories	Categories
- Lack of knowledge and skills on NPMs - Lack of awareness - Not knowing how the methods works	Limited competencies of nurse-midwives on the use of NPMs for managing labour pain	Individual level barriers
- Never considered as important - Lack of childbirth experience - Not using methods frequently - Labour pain management a forgotten practice - Less work experience	Inadequate exposure to labour pain management practices	
- Relieving pain delays a birth - Presence of pain is necessary for birthing - Pain must be there for a woman to give birth	Misconception about labour pain relief	
- Not remembering to be taught - What was learned in college is little - Lacking on the job training	Lack of opportunities for knowledge acquisition	
- Having a constrained number of staff in relation to the high number of clients.	Critical staff shortage amidst large number of clients	Institutional level barriers
- Limited privacy and physical space - Lack of utilities - Lack of pain management guidelines - Limited incentives among nurse-midwives	An unfavourable healthcare facility environment	

Source: In-depth interviews 2020

### Individual level barriers

#### Limited competencies of nurse-midwives on the use of NPMs

Participants expressed that a lack of knowledge and skills about the various NPMs and how they work to relieve pain was a barrier to their use. Additionally, most participants were unaware that they could alleviate or relieve labour pain.

*“I have been working for a long time now*, *but I haven’t seen the methods*, *and I didn’t know whether there are methods to relieve labour pain without the use of drugs ….”****(Participant 11)***.

Furthermore, some participants stated that, despite occasionally using methods like massage and exercises, they never regarded them as pain relief strategies.

*“I didn’t even know if giving a massage to a woman*, *or the way we encourage women to do some simple exercises*, *actually lessens their labour pain …”****(Participant 1)***.

#### Inadequate exposure to labour pain management practices

Participants identified less work experience and a lack of regular practice with labour pain relief approaches as barriers to using NPMs. They admitted that they had never thought of labour pain relief as an important aspect of their service provision. Their responses revealed that nurse-midwives did not use NPMs, such as warm baths, sacral massage, or exercises, in managing labour pain.

*“We have limited skills to use the non-pharmacological methods…. frequently we don’t offer any of the methods to women*, *we simply tell them to tolerate the pain … and I think we never considered it important …”****(Participant 3)***.

Other participants reported that not having gone through the childbirth process may be a barrier to offering labour pain relief to women. Participants perceived that someone who has never experienced the anguish of childbirth may lack the motive to assist women in relieving their pain.

*“If you have never gone through labour pain or haven’t given birth, you cannot feel what women experience when they are in labour, but if you have, you may understand that women need us to be around them and perform a back massage to facilitate a comfortably delivery”****(Participant 4)***.

#### Misconception about labour pain relief

Participants stated misconceptions about labour pain relief as barriers to the use of NPMs. Participants expressed some doubts about using NPMs for labour pain management, which limited their use. They believed that pain is necessary, a sign of good labour progress, and that experiencing pain is essential for timely childbirth. Other participants reported that the labour is not sufficiently painful to relieve; if a woman experiences pain during labour, she likely used local herbs to stimulate labour.

*“The mother benefits from pain during labour because, without it, she may not be progressing well (…) The presence of pain provides reassurance that the foetus is descending, and if the pain decreases, it signifies that the birthing process may be challenging”****(Participant 4)***.

#### Lack of opportunities for knowledge acquisition

The participants said they had never received any training on managing labour pain, neither as part of their professional training nor as continuing education and development for healthcare professionals. They added that the lack of training opportunities in the various NPMs severely limited their experience to the routine use of reassurance and encouragement.

*“We don’t have many training opportunities concentrating on labour pain management, and I haven’t seen anyone teaching it at work yet. I don’t remember receiving instruction on labour pain management during my school days. The existence of training would encourage us to regularly use the labour pain relief methods.”****(Participant 7)***.

### Institutional level barriers

#### Critical staff shortage prevents the use of NPMs in managing labour pain

The participants reported a constrained number of staff in the labour wards while there was an overwhelming number of clients. They stated that the large number of clients and staff shortages often prevent the use of NPMs for the management of labour pain.

*“I typically do it when I have few clients, like two or three, but if they are more than that, I just end up telling a woman to do for herself some methods like the massage, which I do very rarely because of the shortage we have”****(Participant 17)***.

Additionally, participants perceived that the number of clients was too overwhelming and prevented them from using labour pain relief methods. They stated that the increase in the number of women in labour made it challenging for them to provide NPMs to each woman.

*“Often, the challenge is a large number of clients (…) for example, two providers may be caring for eight or ten clients simultaneously (…) it is impossible to provide each client with a back massage”****(Participant 5)***.

#### Unfavourable health facility environment for the use of NPMs

Participants expressed that a lack of a friendly environment discourages the use of NPMs for labour pain relief in the workplace. They mentioned having small labour rooms that are insufficient and unsuitable for using various labour pain relief approaches, such as exercises that require open space. Participants also voiced concerns about the lack of privacy in common labour rooms and the need for modifications suitable for using various approaches for labour pain management.

*“The privacy is also a problem, perhaps because of the setup of our labour wards in many health facilities; to provide a back massage to a woman demands privacy, and sometimes the verbal privacy is important for the woman to feel comfortable as well”****(Participant 6)***.

Participants also reported that the lack of amenities in healthcare facilities, such as water in the restrooms, prevented using warm baths for labour pain relief. They stated that using warm water bathing as an NPM for labour pain relief was a hurdle due to the limited water supply.

*“…water is not always available; it’s on and off (…) There are times when we struggle to get water from other sources, and sometimes water may not be available for the whole day”****(Participant 12)***.

They further asserted that the labour pain management protocols and guidelines constrain the use of NPMs. They added that the facility had various guidelines, protocols and policies, but they lacked aspects of labour pain management.

*“… I have never seen such guidelines for managing labour pain, so perhaps they exist, but our healthcare facilities haven’t received them yet****(Participant 1)***.

Participants reported a lack of financial and non-financial incentives as another barrier to using NPMs for labour pain management. They stated that financial incentives such as extra duty and overtime allowances and non-financial incentives such as recognition and appreciation of nurse-midwives by their managers would boost their morale and encourage them to offer labour pain relief to women.

*“Receiving incentives could potentially motivate me to provide labour pain relief despite my overwhelming workload (…) we work extremely hard until we get headaches; sometimes you find only one skilled birth attendant in an 8-hour shift; you find that you are busy from the beginning to the end of your shift because the number of clients is always high, but the salary remains the same”****(Participant 11).***

## Discussion

We aimed to explore the barriers to using NPMs to manage labour pain by nurse-midwives working in labour wards in two selected district hospitals in eastern Tanzania. Our study findings indicated that the limited competencies of nurse-midwives regarding the use of NPMs, critical staff shortages, and unfavourable environments in healthcare facilities were the main barriers to using NPMs to manage labour pain. Participants highlighted the limited privacy and physical space, lack of utilities, lack of pain management guidelines, and limited incentives constrained the provision of labour pain relief to women.

Our findings show that many NPMs were not widely understood by the nurse-midwives, which hinders their ability to offer them to women. Consistent with our findings, several studies conducted in low- and middle-income countries demonstrate that nurse-midwives have insufficient skills in using NPMs to manage labour pain [1, 26]. Furthermore, our study confirmed what has been documented by other authors: a midwife with less work experience or who conducted fewer deliveries is less likely to demonstrate competence in labour pain management and may lack motivation to do so [20, 44]. In Tanzania, diploma-level and higher education training curricula are competency-based, which contradicts the limited competencies among nurse-midwives revealed by our study [45]. There are three possible explanations for this disparity: inadequate implementation of competence-based training systems, limited hands-on exposure for students, or insufficient use of NPMs for labour pain management by senior healthcare providers.

Our findings demonstrate that nurse-midwives perceive labour pain as a normal event that requires tolerability rather than management, which prevents the use of labour pain relief techniques. Other studies from developing countries also report this finding [19, 20, 46]. The latter studies showed that people regarded labour pain as a natural process that women should learn to cope with it. These perceptions are in contrary to what has been reported in developed countries, where labour pain management is a fundamental aspect of intrapartum care and women have the autonomy to choose their preferred pain relief method [47].

The healthcare providers’ attitudes and beliefs towards labour pain management may affect the use of NPMs to manage labour pain [18, 19, 48]. In our study, participants misconceived the idea of relieving labour pain and believed that the pain is necessary for a successful birth. This negative attitude towards labour pain contributed to not using the NPMs to manage labour pain. Accordingly, obstetrics healthcare providers with a good attitude are more likely to use NPMs for labour pain management compared to those with a negative attitude [19, 49]. These findings demonstrate the necessity of moulding the attitudes of nurse-midwives and obstetricians through continued professional development and mentorship to help mitigate this challenge.

Our findings indicate that a heavy workload amidst a staff shortage challenged the provision of NPMs to manage labour pain. This finding conforms to what Tanzania has documented: the country has a critical shortage of healthcare workers, impacting the delivery of quality care. [50, 51]. The effect of a shortage of staff in the healthcare system on the quality of care, including the provision of NPMs to women for labour pain relief, is not unique to Tanzania [1, 20]. The Matlala and Lumadi study directly links the shortage of medical personnel, particularly nurse-midwives, to poor quality care delivery due to increased workload, leading to low morale and burnout [52].

Our study findings revealed that the insufficiency of supporting utilities in delivery rooms, including water and showers, a lack of privacy, and physical space restricted the application of NPMs. The availability of supportive infrastructure could enable nurse-midwives to instruct women to walk, squat, take showers, or even bring a birth companion. Literature shows that the unfavourable working conditions in healthcare facilities, which are characterized by substandard infrastructure, can be a significant barrier to using NPMs for labour pain management [46, 53]. Ensuring the appropriate set-up and design of delivery rooms is crucial to facilitating the implementation of labour pain relief techniques and, consequently, delivering high-quality childbirth care.

To facilitate a humanized childbirth experience, the Tanzanian respectful maternity care guideline recommends that every woman receive respectful care, continuous emotional support, and a companion of her choice [54]. Conversely, our study revealed that nurse-midwives lacked knowledge of NPMs for labour pain management due to their unfamiliarity with labour pain management guidelines. The absence of appropriate guidelines and policies influences healthcare providers not to use NPMs to manage labour pain [2, 10]. Pre-service training, on-the-job training, and provider policies and guidelines all provide essential knowledge that substantiates the implementation of labour pain management techniques during childbirth in healthcare facilities [49]. This barrier may be partly mitigated by revisiting policies and guidelines to incorporate labour pain relief measures as an essential element of quality of care.

### Implications to nursing practice, education and research

This study provides an in-depth description from nurse-midwives of important barriers to address for labour pain management as part of the care provided to women during labour. Uncovering the barriers that nurse-midwives face to using NPMs provides evidence that improving nursing and midwifery knowledge and skills is key to enhancing the quality of care provided during childbirth. Promoting the uptake of NPMs is dependent on improved healthcare facility infrastructure, the availability of guidelines and policies, and having an adequate number of midwives. Moreover, nurse-midwives should receive in-service training on using NPMs for managing labour pain, cultivating positive attitudes towards using NPMs, and advocating for including NPMs in nursing and midwifery curricula. In addition, promoting the use of NPMs for labour pain management helps women cope with pain without relying on medications, feel more in control of their labour and birthing process, and thus have a positive childbirth experience. To develop measures to encourage the use of NPMs, a mixed-methods research study analyzing their practice and the reason for their low uptake is essential.

### Study strengths and limitations

Although we enhanced the methodology rigor of our study through Lincoln and Guba`s four criteria —credibility, dependability, transferability and confirmability [55], it is not without limitations. The major limitation of our study was the social desirability effect. The study lead was a nurse-midwife, potentially leading participants to respond in favour of the researcher’s expectations. However, we adopted different strategies to offset this limitation. Firstly, we triangulated the midwife researcher with other researchers who were not midwives during the conduct of the interviews. Secondly, the participants ranged from junior to senior midwives, and the team lead, who was in her mid-career, provided a comfortable speaking environment for these diverse groups. Third, adopting saturation within and across facilities ensured adequate participants to offset social desirability. Finally, the research team’s vast experience ensured that study participants received an adequate explanation of the study’s objective before the interviews began.

## Conclusions

Most of the barriers to the use of NPMs for managing labour pain are within the health system’s capacity to address. Strengthening the training of midwives during professional training and through continued professional development has the potential to address the competence gap. In contrast, mentorship from senior colleagues on using NPMs can address the misconceptions about the use of NPMs. Government decisions through the Ministry of Health on prioritizing labour pain management as an area of appropriate investment are necessary due to staff shortage and infrastructure challenges. Further studies should focus on the community-level barriers to the uptake of NPMs among pregnant women, as our study only collected provider perspectives.

## Data Availability

The data analysed during the current study are available from the corresponding author upon reasonable request.

## Abbreviations

CEmONC: Comprehensive Emergency Obstetric and Newborn Care
NPMs: Non-Pharmacological Methods
SIDA: Swedish International Development Cooperation
TENS: Transcutaneous Electrical Nerve Stimulation
